# Prevalence and risk factors associated with precancerous cervical lesions among women in two cities in Cameroon

**DOI:** 10.11604/pamj.2022.41.276.21972

**Published:** 2022-04-06

**Authors:** Bernard Wabo, Dickson Shey Nsagha, Théophile Njamen Nana, Clement Jules Nguedia Assob

**Affiliations:** 1 Department of Medical Laboratory Science, Faculty of Health Sciences, University of Buea, Buea, Cameroon,; 2 Department of Public Health and Hygiene, Faculty of Health Sciences, University of Buea, Buea, Cameroon,; 3 Department of Obstetrics and Gynaecology, Faculty of Health Sciences, University of Buea, Buea, Cameroon

**Keywords:** Precancerous cervical lesions, Pap smear, prevalence, risk factors

## Abstract

**Introduction:**

cervical cancer is the fourth commonest cancer of women world-wide with increasing incidence in developing countries. This study determined the prevalence and assessed risk factors associated with precancerous cervical lesions among women in Cameroon.

**Methods:**

this cross-sectional study enrolled 925 women participants of a screening campaign for precancerous cervical lesions from June to November 2018 in the selected hospitals. A convenience sampling technique was used and socio-demographic, sexual and reproductive data collected from consented participants by means of self-administered questionnaire. During the gynaecologic examination, a cervical smear was collected, stained by the Papanicolaou staining technique and the results classified according to the Bethesda 2014 guidelines. Frequency, percentage, Chi square and regression analysis were conducted using SPSS version 20 and p-value considered at 0.05.

**Results:**

of the 925 participants aged 25-65 years (mean 40.2±10.2 SD), 113 (12.2%) had the lesions among whom 9 (7.9%) had atypical squamous cells of undetermined significance, 75 (66.4 %) had Low-grade squamous intraepithelial lesion and 29 (25.7%) had high-grade squamous intraepithelial lesion. Factors associated with the lesions were: age 1.85 [1.42-2.41; p= 0.001] and parity [OR= 1.46; 95% CI: 1.30-1.89; P= 0.004].

**Conclusion:**

the prevalence of precancerous lesions was 12.2%. Age, parity were significant risk factors. Regular screening targeting the population at risk in this study becomes a priority.

## Introduction

Cervical cancer is a worldwide major concern [1]. Mortality due to cervical cancer has been suggested to be an indicator of health inequities, since 86% of all deaths due to cervical cancer are in developing, low- and middle-income countries [2]. Cervical cancer is a major cause of morbidity and mortality among women in poor resource settings, especially in Africa [3]. In Cameroon, cervical cancer is a major public health issue with the national prevalence estimated at 3.9 % among women aged 27-55 years in 2013 [4]. About 99.7 % cases of cervical cancer are due to *Human Papillomavirus* (HPV), particularly types 16 and 18 which are responsible for more than two-thirds of all precancerous cervical lesions and cervical cancers [5]. Younger age, earlier age of the first pregnancy, oral contraception, early sexual activities and higher number of children born are all risk factors for cervical cancer [6]. Asymptomatic precancerous cervical lesions can go for many years which in most cases, might regress spontaneously and only a small number of them progressing to invasive cancer [7]. Unlike in developed countries were the incidence and mortality of this cancer have decreased significantly because of the efforts made to detect precancerous cervical lesions early, the disease still spread unknown among women in developing countries [8]. Cervical cancer is preventable through screening; early detection and treatment of pre-invasive cervical lesions [9]. Several screening modalities are available for the detection of cervical cancer and its precursor lesions. Those tests include: cytology or Papanicolaou (Pap) testing, visual inspection using acetic acid (VIA) or Lugol´s iodine (VILI), and HPV-test [10]. Ndenkeh *et al*. (2018) reported that the inability of the National Health Information System (NHIS) to make available updated data on the incidence and mortality of cancer was responsible to the increase prevalence of cervical cancer in Cameroon [11]. Our objective was to determine the prevalence and risks associated with precancerous cervical lesions of the cervix in women in four of the five reference hospitals in Douala and Yaoundé, respectively the economic and political capital of Cameroon with a high and cosmopolitan population.

## Methods

**Study design:** a cross-sectional study conducted between June and November 2018 enrolled sexually active/asymptomatic women aged 25-65 years sensitized for a free cervical cancer screening campaign in four hospitals in Yaoundé and Douala, Cameroon.

**Study settings and populations:** four reference hospitals were purposely selected for the study. The reasons for their selection were the availability of facilities for cervical cancer screening and an average of at least two hundred patients flow per month at the gynaecologic unit. From Yaoundé, two study sites were selected namely the Yaoundé University Teaching Hospital (YUTH) and the Yaoundé Gynaeco-Obstetric and Paediatric Hospital (YGOPH) and from Douala were the Douala General Hospital (DGH) and Douala Gynaeco-Obstetric and Paediatric Hospital (DGOPH). Participants were patients or their relatives visiting the selected hospitals or women sensitized for the cancer screening campaign via media messages and banners display in front of the hospital. Sexually active/ asymptomatic volunteers aged 25-65 years who consented to participate in the study were included. Women With: 1) prior hysterectomy; 2) prolonged menstruation; 3) pregnancy/in the post-partum period; 4) existing precancerous/cancerous lesions; 5) a history of gynaecologic malignancies or 5) pelvic radiation were excluded.

**Administrative and ethical considerations:** the administrative clearance was obtained from the Dean of the Faculty of Health Sciences, University of Buea (Cameroon) and the authorisation for sample collection from the respective hospital administration. The study was also approved by the Ethical Committee of the Faculty of Heath Sciences- Institutional Review Board, University of Buea, South west Region of the country (N° 2018/0254/UB/SG/IRB/FHS). The aim of the study was explained to participants; who were requested to fill in a structured questionnaire containing questions on their socio-demographic characteristics, sexual and reproductive histories. Anonymity and confidentiality were respected. A verbal/written informed consent was obtained from each of the participants.

**Sampling methods:** a minimum sample size of 175 was obtained using the Lorentz formula [12].


N=Z2P(1−P)e2

Where Z= 1.96 at 95% confidence interval), e: margin of error 5%, P: expected proportion of cervical cancer in Cameroon 12.7 % [13]. In order to have enough sample size necessary for the accuracy of values and generalization of results, we purposely enrolled 925 participants corresponding to the combined average of 2 115 monthly patients flow in the study sites. They were proportionally distributed as followed: in the Douala Gynaeco-Obstetric and Paediatric Hospital 175 (18.9%) samples from 400 visitors, in the Douala General Hospital 256 (27.7%) samples from 586 visitors, in the Yaoundé University Teaching Hospital 223 (24.1%) samples from 510 visitors and in the Yaoundé Gynaeco-Obstetric and Paediatric Hospital 271 (29.3%) samples from 620 visitors. Participants consecutively enrolled for the study.

**Procedures:** participants were invited to lie in a lithotomy position and a sterile disposable speculum inserted in the vagina to visualise the cervix for any abnormalities such as the quantity of the vagina discharge, cervicitis, ectropion, nabothian cyst, ulceration. A gynaecologist supervised all gynaecologic procedures. A cervical smear was then collected using Ayre spatula and cytobroom, immediately fixed, transported to the laboratory and later stained by the Papanicolaou staining technique [7]. The microscopic examination of the slides were blindly made by two experienced cytologists and confirmed by a pathologist. The results of the Pap test were classified using the Bethesda 2014 guidelines [14] as normal, atypical squamous cells of undetermined significance (ASC-US), Low-grade squamous intraepithelial lesion (LSIL) and High-grade squamous intraepithelial lesion (HSIL).

**Data management and analysis:** data collected using questionnaire and laboratory form were daily recorded in a log book to make up the data source, verified for consistency, completeness and later computerized on Microsoft Excel 2010. Data were analysed using Statistical Package for Social Sciences (SPSS) version 20. Descriptive statistics: mean and standard deviation were used for continuous variables and percentage for categorical variables. The chi-square test was used to assess the association between categorical variables. Bivariate and multivariate regression analyses were used to assess independent variables in the prediction of precancerous cervical lesions. The statistical significance was considered at 0.05.

## Results

**Distribution of participants:** of the 955 women contacted for the screening, 925 aged 25-65 years (mean 40.2± 10.2 SD) were enrolled. The response rate was 96.85 %. Of the 925 participants, 271 (29.3%) were from the YGOPH, 256 (27.6%) from the DGH, 223 (24.1%) from the YUTH and 175 (18.9%) from the DGOPH.

**Prevalence of precancerous cervical lesions:** we recorded 113 (12.2%) cases of positive Pap smear. Among positive cases, we had 9 (7.9%) ASCUS, 75 (66.4%) LSIL and 29 (25.7%) HSIL. Positive cases were further distributed according to the health facilities as followed: 36 (31.8%) for the YUTH, 35 (31.0%) for the YGOPH, 28 (24.8%) for the DGH and 14 (12.4%) for the DGOPH.

**Prevalence of the lesions according to the socio-demographic characteristics:** on Table 1, we reported the following major characteristics: 471 (50.9%) women aged 25-38 years, 565 (61.1%) married, 327 (35.4%) with salary jobs, 432 (46.7%) with the secondary school education. About their behavioural background, Table 1 equally shows that 850 (91.9%) participants were no-smokers and 11 (1.2%) were permanent smokers. In addition, 692 (74.5%) of them were occasional alcohol consumers, 59 (6.4%) permanent consumers and 174 (18.8%) were no-alcohol consumers. Regarding the contraceptive methods used, 344 (37.5%) used oral contraceptive occasionally and 592 (64.0%) used condom inconsistently (Table 1). Table 1 also shows 47 (41.6%) and 37 (32.7%) participants with precancerous cervical lesions aged 39-52 and 25-38 years respectively. There was a statistically significant difference between age and precancerous cervical lesions (p= 0.001). High prevalence of the lesions in other categories was as followed: 72 (63.7%) cases for those married, 42 (37.2%) cases for housewives and 54 (47.8%) cases for those with the secondary school education. There was no statistically significant difference between marital status (p = 0.180), profession (p= 0.472), education (p= 0.339) and precancerous cervical lesions. Regarding the behavioural factors, we obtained 105 (11.4%) no-smokers and 94 (83.2%) casual alcohol consumers with the lesions. Again, there was no statistically significant difference between smoking (p= 0.461), alcohol consumption (p= 0.085) and precancerous cervical lesions (Table 1).

**Table 1:** prevalence of precancerous cervical lesions according to socio-demographic factors

Socio-demographic characteristics	Total [n=925, (%)]	Positive Pap test [(n=113, (%)]	Chi-square	P-value
**Age range (years)**			22.75	0.001
25-38	471 (50.9)	37 (32.7)		
39-52	325 (35.1)	47 (41.6)		
53-65	129 (13.9)	29 (25.7)		
**Marital status**			4.89	0.180
Married	565 (61.1)	72 (63.7)		
Singles	284 (30.7)	27 (23.9)		
Widowed	58 (6.3)	11 (9.7)		
Divorced	18 (1.9)	3 (2.7)		
**Occupation**			2.51	0.472
Housewives	285 (30.8)	42 (37.2)		
Salary jobs	327 (35.4)	36 (31.9)		
Traders	150 (16.2)	16 (14.1)		
Unemployed	163 (17.6)	19 (16.8)		
**Education level**			3.36	0.339
Informal	21 (2.3)	1 (0.9)		
Primary	132 (14.3)	21 (18.6)		
Secondary	432 (46.7)	54 (47.8)		
Tertiary	340 (36.8)	37 (32.7)		
Smoking			1.55	0.461
Permanent	11 (1.2)	0 (0.0)		
Casual	64 (6.9)	8 (7.1)		
Never	850 (91.9)	105 (92.9)		
**Alcohol consumption**			4.93	0.085
Permanent	59 (6.4)	4 (3.5)		
Casual	692 (74.8)	94 (83.2)		
Never	174 (18.8)	15 (13.3)		

**Prevalence of precancerous cervical lesions according to the sexual characteristics:** Table 2 shows that, 596 (64.4%) women had their first menses at age less than 15 years, 136 (14.7%) at age greater than 15 years and 193 (20.9%) other did not remember the year. The age at first menses ranged from 8-19 years (mean 11.02 ± 5.87 SD). Our data also show that, 668 (72.2%) had initiated sexual activity at age greater than 15 years while 198 (21.4%) other could not remember the year. The age at first sex ranged from 12-31 years (mean: 14. 66± 8.04 SD). Table 2 again reveals that 721 (77.9%) participants declared less than 3 sexual partners in the pass one year also, 445 (48.1%) other declared they had more than 3 sexual acts every month. Following the Pap test, we recorded the lesions in 70 (61.9%) cases among participants who declared having their first menses at the age less than 15 years (p= 0.458), in 83 (73.4%) other who declared having first sex at age greater than 15 years (p= 0.951) and in 86 (76.1%) other who affirmed having less than 3 sexual partners in the past one year (p=0.300). There was almost equal distribution of precancerous cervical lesions between having less than 3 sexual acts or more monthly (p= 0.172) (Table 2).

**Table 2:** prevalence of precancerous cervical lesions according to the sexual history

Sexual history	Total [n=925, (%)]	Positive Pap test [n=113, (%)]	Chi-square	P-value
**Age at first menses(years)**			1.56	0.458
15	596 (64.4)	70 (61.9)		
>15	136 (14.7)	21 (18.6)		
I do not remember	193 (20.9)	22 (19.5)		
**Age at first sex(years)**			0.100	0.951
15	59 (6.4)	7 (6.2)		
>15	668 (72.2)	83 (73.4)		
I do not remember	198 (21.4)	23 (20.4)		
**Number of sex partners**			2.41	0.300
3	721 (77.9)	86 (76.1)		
> 3	109 (11.8)	11 (9.7)		
I do not remember	95 (10.3)	16 (14.2)		
**Number of sex act/month**			3.52	0.172
3	351 (37.9)	39 (34.5)		
> 3	445 (48.1)	40 (35.4)		
I do not remember	129 (13.9)	34 (30.1)		

**Prevalence of precancerous cervical lesions according to the reproductive factors:** regarding their reproductive variables, 9 (1.00%) participants had their first pregnancy at the age less than 15 years, 787 (85.1%) other at age greater than 15 years. About 129 (13.9%) declared no previous pregnancy. The age at first pregnancy ranged between 13-43 years (mean: 18.9±8.6 SD) (Table 3). We also recorded that, 195 (21.1%) participants had more than five parities, 197 (21.3%) were menopause and 579 (62.6%) reported previous abortion. Finally, our results revealed 68 (7.4%) HIV positive cases among whom 62 (91.2%) were on anti-retroviral therapy (Table 3). While assessing the relationship between reproductive variables and precancerous cervical lesions, our results show that, of the 113 participants with positive Pap smear, 100 (88.5%) had their first pregnancy at age greater than 15 years (p= 0.043), 52 (46.0%) had 3-5 parities (p= 0.009) and 68 (60.2%) were within the reproductive age (p= 0.001). There was an equal distribution of the lesions between participants who declared [32 (28.3%)] voluntary and spontaneous abortion [33 (29.2%)] (Table 3)]. Behavioural factors analysed revealed that, of the 113 participants with positive Pap smear, 75 (66.4%) do not use oral contraceptive (p= 0.403) likewise, 61 (54.0%) do not condom during sexual intercourse (p= 0.051). We and lastly, found 12 (10.6%) cases with the lesions to be HIV positive (p= 0.159) (Table 3).

**Table 3:** prevalence of precancerous cervical lesions in relation to the reproductive history

Reproductive history	Total [n=925, (%)]	Positive Pap test [n=113, (%)]	Chi- square	P-value
**Age at first pregnancy**			6.32	0.043
≤15	9 (1.00)	3 (2.7)		
>15	787 (85.1)	100 (88.5)		
Never pregnant	129 (13.9)	10 (8.8)		
**Parity**			9.43	0.009
<2	366 (39.6)	30 (26.5)		
3-5	364 (39.4)	52 (46.0)		
> 5	195 (21.0)	31 (27.4)		
**Menopause**			26.35	0.001
Yes	197 (21.3)	45 (39.8)		
No	728 (78.7)	68 (60.2)		
**Abortion**			1.56	0.669
Voluntary	304 (32.8)	32 (28.3)		
Spontaneous	235 (25.4)	33 (29.2)		
Both	40 (4.3)	5 (4.4)		
No abortion	346 (37.4)	3 (38.0)		
**Use of oral contraceptive**			4.47	0.214
Never	581 (62.8)	75 (66.4)		
casual	289 (31.2)	30 (26.5)		
Permanent	34 (3.7)	7 (6.2)		
Former	21 (2.3)	1 (0.9)		
**Use of condom**			5.94	0.051
Consistent	53 (5.7)	7 (6.2)		
Inconsistent	592 (64.0)	45 (39.8)		
Never	280 (30.3)	61 (54.0)		
**HIV status**			2.02	0.159
Yes	68 (7.4)	12 (10.6)		
No	857 (92.6)	101 (89.4)		

**Socio-demographic risk factors associated with precancerous cervical lesions**: there was a statistically significant association between age and precancerous cervical lesions [OR=1.85; 95% CI: 1.42-2.41; p= 0.001]. Participants aged between 25-38 years were 3.4 times [OR=3.40; 95%CI: 1.99-5.80; p= 0.001] more likely to have the lesions than those aged between 39-52 years [OR=1.72; 95% CI: 1.02-2.88; p= 0.040] (Table 4). The marital status was weakly associated with precancerous cervical lesions [OR= 1.10; 95%CI: 0.86-1.37; p= 0.460]. But, participants who declared being single were 2.23 times [OR= 2.23; 95% CI: 1.03-4.76; p= 0.041] more likely to have the lesions than those who were divorced [OR= 1.17; 95% CI: 1.07-4.80; p=0.826] and those married [OR= 1.60, 95% CI: 0.80-3.23; 0.188]. Similarly, there was a weak association between smoking and precancerous cervical lesions [OR= 1.18; 95% CI: 0.55-2.56; p= 0.661] (Table 4).

**Table 4:** analysis of socio-demographic risk associated with precancerous cervical lesions

Socio-demographics	Bivariate OR(95%CI)	P-value	Multivariate OR(95%CI)	P-value
**Age (years)**				
25-38	1.85 (1.42-2.41)	0.001	3.40 (1.99-5.80)	0.001
39-52			1.72 (1.02-2.88)	0.040
53-65			1.00	-
**Marital status**	1.10 (0.86-1.37)	0.460		
Married			1.60 (0.80-3.23)	0.188
Single			2.23 (1.03-4.76)	0.041
Widowed			1.00	-
Divorced			1.17 (1.07-4.80)	0.826
**Occupation**	0.90 (0.77-1.05)	0.201		
Housewives			0.97 (0.57-1.63)	0.906
Salary jobs			1.00	-
Traders			1.22 (0.64-2.34)	0.555
Unemployed			0.69 (0.37-1.30)	0.249
**Education level**	0.90 (0.76-1.05)	0.193		
Informal			0.33 (0.04-2.60)	0.294
Primary			0.24 (0.03-1.88)	0.172
Secondary			0.30 (0.04-2.31)	0.247
Tertiary			1.00	-
**Smoking**	1.18 (0.55-2.56)	0.661		
Ever			1.28 (0.57-2.85)	0.545
Never			1.00	-
**Alcohol consumption**	0.83 (0.55-1.25)	0.390		
Permanent			1.37 (0.41-4.18)	0.603
Casual			0.60 (0.33-1.06)	0.080
Never			1.00	-

**Reproductive risk factors associated with precancerous cervical lesions**: regarding the reproductive history of participants, parity was associated with 1.46 times increased risk of precancerous cervical lesions [0R= 1.46; 95%CI: 1.13-1.89, p= 0.004]. Participants with less than two parties were 2.12 times more likely to have precancerous cervical lesions [OR=2.12; 95% CI: 1.23-3.61; p = 0.006] compared to those with 3-5 deliveries [OR= 1.13: 95% CI: 0.70-1.84; p= 0.610] (Table 5).

**Table 5:** assessment of reproductive risk associated with precancerous cervical lesions

Reproductive history	Bivariate OR(95%CI)	P-value	Multivariate OR(95%CI)	P-value
**Use of oral contraceptive**	1.10 (0.87-1.32)	0.503		
Casual			1.11 (0.68-1.77)	0.690
Permanent			0.59 (0.24-1.46)	0.255
Former			3.72 (0.47-29.32)	0.212
Never			1.00	-
**Use of condom**	1.09 (0.88-1.34)	0.404		
Consistent			1.00	-
Inconsistent			1.32 (0.57-3.06)	0.511
Never			0.79 (0.33-1.87)	0.599
**Age at first pregnancy (years)**	0.88 (0.65-1.20)	0.438		
≤ 20			0.56 (0.27-1.14)	0.109
≥ 21			0.57 (0.28-1.15)	0.119
Never pregnant			1.00	-
**Parity**	1.46 (1.30-1.89)	0.004		
0-2			2.12 (1.23-3.61)	0.006
3-5			1.13 (0.70-1.84)	0.610
≥ 6			1.00	-
**Abortion**	1.00 (0.85-1.12)	0.925		
Spontaneous			0.97 (0.57-1.63)	0.909
Voluntary			1.19 (0.70-2.01)	0.520
**Both**			1.18 (0.40-3.33)	0.771
No abortion			1.00	-
**HIV status**	-	-		
Yes			0.62 (0.30-1.27)	0.195
No			1.00	-

## Discussion

Cervical cancer is a major public health concern in the 21^st^ century [15]. The current study was conducted to determine the prevalence and to assess risk factors associated with precancerous cervical lesions among women in four reference hospitals in Douala and Yaoundé cities of Cameroon.

**Prevalence of precancerous cervical lesions:** the overall prevalence of precancerous cervical lesions was 12.2%. Abnormalities recorded were ASCUS (7.9%), LSIL (66.4%) and HSIL 25.7%). There are geographic variations in the prevalence of cervical cancer. It was found to be; 12.7% in the Northern Cameroon, 29.1% and 13.8% in Yaoundé, and 16.9% among women on antiretroviral therapy [13,16-18]. High prevalence was located in hospitals of the Centre region which was in accordance with the findings of Tebeu *et al*., (2013). Disparities in regional distribution of the prevalence of precancerous cervical lesions might be attributed to differences in the socio-behavioural characteristics of the populations, the availability of the screening centres and to the accuracy screening test used.

**Risk factors associated to precancerous cervical lesions:** we reported a strong association between age and precancerous cervical lesions. Participants of 25-38 years were more likely to have the lesions than those in the other age categories. Our finding was in variance with the report of a higher odd of developing cervical precancerous cervical lesions in women aged 40-49 years [19]. Increased risk of precancerous cervical lesions in the lower age group in this study might be related to the acquisition of HPV during unsafe sexual intercourse at early age [20]. Our data also showed an increased risk of precancerous cervical lesions among single than among married and divorced women. In contrary, Antic *et al*., (2014) in a study of the differences in risk factors for cervical dysplasia in Serbia reported that living in marriage reduced the risk for cervical cancer development [21]. Finding most single women in our community more likely to have precancerous cervical lesions could be due to their predisposition for multiple sexual partners, a factor which was identified to be significantly associated with SIL [22]. Multiple sexual partnership in most communities could link to the low socio-economic status favouring sexual promiscuity. Reis *et al*. (2011) in a case control study reported no association between profession, education and cervical cancer [23]. But from this study, trading represented to some extend a risk of precancerous cervical lesions though with no statistically significant difference. Considering that weak association, we anticipated that, women traders, by the nature of their profession, are more likely to have multiple sexual partners and unsafe sexual intercourse. This study suggested that high education level was protective for precancerous cervical lesions. The justification supporting such observation could be that high education level increases knowledge on risk factors and screening practice of cervical cancer [24]. Raising women´s education level in our setting is challenging since most high school in Africa are limited to urban areas.

There was no association between cigarette smoking and precancerous cervical lesions. Our finding contradicted the report that cigarette smoking, both active and passive increases the risk of precancerous lesions [25]. Given the controversies about the issue, an author documented that the association between cigarette smoking and risk of precancerous cervical lesions is causal and time dependent [26]. That lack of association could be justified by the finding of few cigarette smokers among our participants. That apparent reduction in the number of smokers might be the result of the active campaigns against tobacco smoking and drugs consumption organized by the Cameroon Ministry of Health, private institutions and Non-governmental organizations. Alcohol consumption is frequent among wen in Cameroon but, this study revealed the majority of participants to be casual consumers. Permanent alcohol consumption increased the risk of precancerous cervical lesions by 1.37 times in this study. Even though that association was no statistically significant, our findings were in accordance with the suggestions by an author that chronic alcohol abuse, acute and moderate alcohol consumption can adversely affect the immune system with subsequent increase susceptibility to chronic infections [27]. Oral contraceptive is often used by women against unwanted pregnancy. The current study found a low risk of precancerous cervical lesions among participants who had ever taken an oral contraceptive. Kassa (2018) in a case control study also found increase risk of precancerous lesions among oral contraceptive users in Ethiopia [28]. The role of oral contraceptive in the initiation of precancerous cervical lesions has been subject of controversies [27]. Author who ascertained that association suggested that, using hormonal contraception for 1-4 years and 5-25 years increases the risk of cervical cancer to 2.0 and 4.5 times respectively [29]. From the above, we can conclude that, the association is duration dependent.

Both male and female condoms are mechanical barriers used to prevent unwanted pregnancy. The use of condom has been subject of many controversies. In the current study, condom was associated with reduced risk of precancerous cervical lesions; that was in line with the report suggesting the use of condom to be beneficial in protecting against sexually transmitted infections but, a different study documented the use of condom in the last sexual intercourse to be a predictor of HPV infection [30,31]. Consistent use of condom might further reduce the risk of HPV infection in Cameroon since the majority of those with the precancerous cervical lesions declared inconsistent use of condom during sexual intercourse. There are controversies on whether HIV-positive women are more likely to develop cervical cancer than HIV-negative women [32]. Previous study demonstrated that the detection of HPV in HIV-infected women is a reflection of either reactivation or persistence of pre-existing HPV infection rather than recent HPV acquisition [33]. Finding no association between HIV and precancerous cervical lesions in this study could be due to the fact that HIV positivity was based on declaration by participants and did not reflect the HIV status of the study population.

Age at first menses, age at first sex and age at first pregnancy are sequentially related steps in women reproductive life, with no statistically significant association with precancerous cervical lesions in the current study. That lack of association contradicted previous study [6]. That lack of association could be supported by the finding in our data that most participants were mature before sexual debut and first pregnancy. Participants who declared less than two parities in this study were 2.12 times more likely to develop precancerous cervical lesions than those with greater number. This finding was in variance with the report that multiparity was a significant risk for the development of cervical cancer [4,34]. Jensen *et al*. (2013) in a cohort-study among high-risk HPV positive women reported that childbirth but not pregnancy was predictive of cervical interstitial neoplasia [35]. For that reason, a study found a significant association between the number of deliveries and abortions as well as of deliveries in adolescent age with the cervical cancer [21]. The association between parities of less than two and precancerous cervical lesions could be due to cervical trauma during childbirth, abortion and eventually hormonal status during pregnancy which might facilitate HPV acquisition or persistence [36].

## Conclusion

From the current study, the prevalence of precancerous lesions was 12.2%. The LSIL was most frequent. The Hospitals in Yaoundé had the highest prevalence. Risk factors associated with precancerous cervical lesions were: age and parity. Smoking, use of oral contraceptive and abortion were not statistically significant risk factors for precancerous cervical lesions. Regular screening targeting the population at risk in this study becomes a priority.

### What is known about this topic

- Cervical cancer is a public health in developing countries;
- Pap test, a common screening test can contribute to reduce the incidence and mortality due to cervical cancer;
- Human papilloma viruswhich is the main cause of cervical cancer is transmitted sexually, cervical cancer if diagnosed early is curable.

### What this study adds

- The prevalence of precancerous cervical lesions is relatively high in Cameroon;
- Women aged 25-38 years and those with less than three deliveries are likely to develop precancerous cervical lesions;
- High education level contribute to reduced risk of exposure to HPV which causes cervical cancer.
